# University and Hospital Bulletin

**Published:** 1904-09

**Authors:** 


**A Monthly Review of Medicine and Surgery.**

*EDITOR:*

WILLIAM WARREN POTTER, M. D.

All communications, whether of a literary or business nature, books for review and exchanges should be addressed to the editor: 284 Franklin Street, Buffalo, N. Y.

Vol. Xliv.—Lx. SEPTEMBER, 1904. No. 2

### University and Hospital Bulletin.

THE Journal in its next issue will inaugurate a new section, to be known as the *"University and Hospital Bulletin,"* which is to be devoted to the interests of the medical department of the university particularly, and the hospitals of the city in general, in so far as the work in them is connected with medical education. It is the intention to make this section an interesting and public-spirited supplement to the Journal, in which shall be found as nearly a perfect record of university advancement as it is possible to give from month to month.

While, as a matter of course, questions of purely scientific character will be dealt with, they will be given in narrative form—the strictly technical paper and its discussions being reserved for the other pages of the Journal. Opinions of a general character will not be expressed; but an effort will be made to present and maintain a strictly news department devoted to the University of Buffalo and the fostering and upbuilding of its interests. Clinical hospital work by university leaders will be given in what may properly be called serial form. Unusual cases in hospital practice and in charge of men who have no university connection, will be given space commensurate with the importance of the case, the ingenuity of its treatment or the rarity of its features.

As near as may be all departments will be represented from time to time; but standing features will include surgery, medicine and pathology, the general news items of the medical department, hospital clinics, the work of the students and the fraternities. The faculty of the medical department will act as an advisory editorial staff of this new division, which, we believe, will prove of great interest to the alumni and students and we hope it will become of distinct benefit to the University of Buffalo and the hospitals of the city.

